# Envejecimiento y cuidados a largo plazo en Chile: desafíos en el contexto de la OCDE

**DOI:** 10.26633/RPSP.2017.86

**Published:** 2017-07-20

**Authors:** Pablo Villalobos Dintrans

**Affiliations:** 1 Harvard University, T H Chan School of Public Health Boston, Massachusetts, Estados Unidos de América Unidos de América Harvard University, T H Chan School of Public Health, Boston, Massachusetts, Estados Unidos de América

**Keywords:** Cuidados a largo plazo, envejecimiento de la población, actividades cotidianas, economía, Organización para la Cooperación y el Desarrollo Económico, Chile, Long-term care, demographic aging; activities of daily living, economics, Organisation for Economic Co-operation and Development, Chile, Assistência de longa duração, envelhecimento da população, atividades cotidianas, economia, Organização para a Cooperação e Desenvolvimento Econômico, Chile

## Abstract

*Chile se encuentra en pleno proceso de transición demográfica y su población envejece rápidamente. Esta situación presenta múltiples desafíos de política pública, incluidos los del área de la salud pública. En concreto, la relación entre el envejecimiento y la pérdida de la autonomía exige diseñar con urgencia una política de cuidados a largo plazo en el país. El objetivo de este documento es describir el escenario actual de los cuidados a largo plazo en Chile usando la experiencia de los países de la Organización para la Cooperación y el Desarrollo Económico, para poner de manifiesto la necesidad de avanzar en el diseño y el financiamiento de una política coordinada en el país, que permita afrontar con antelación los desafíos del envejecimiento en las próximas décadas*.

## Abstract (en)

Chile is fully in the process of demographic transition, with a rapidly aging population. This situation poses multiple public policy challenges, including those in the public health sector. Specifically, the association between aging and the loss of autonomy calls for the rapid design of a long-term care policy in the country. The purpose of this article is to describe Chile’s current situation with respect to long-term care in aging, using the experience of the countries of the Organisation for Economic Co-operation and Development to draw attention to the need to move forward with the design and financing of a coordinated policy in the country that will permit early action to meet the challenges of aging in the coming decades.

## RESUMO

O Chile está em pleno processo de transição demográfica e a população do país está envelhecendo rapidamente. Esta situação apresenta vários desafios de políticas públicas, inclusive em saúde pública. Especificamente, a relação entre o envelhecimento e a perda de autonomia requer o planejamento com urgência de uma política de assistência a longo prazo no país. O objetivo deste documento é descrever o cenário atual de assistência a longo prazo diante do envelhecimento no Chile com base na experiência dos países da Organização para a Cooperação e Desenvolvimento Econômico a fim de evidenciar a necessidade de avançar no planejamento e financiamento de uma política coordenada que permita enfrentar com antecedência os desafios do envelhecimento no país nas próximas décadas.

**FIGURA 1.**
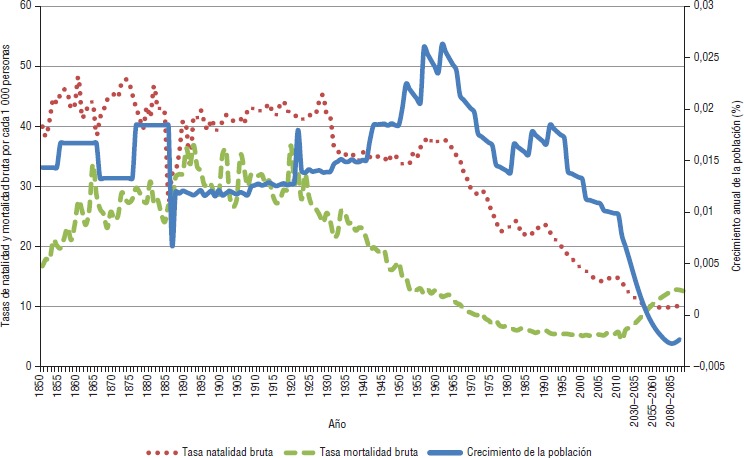
Tasas de crecimiento, natalidad y mortalidad en Chile, 1850–2100

Chile está en pleno proceso de transición demográfica conforme al patrón clásico descrito en la bibliografía (1–3): una primera etapa marcada por un descenso de la mortalidad y crecimiento poblacional, seguida por una fase de reducción de la fertilidad y crecimiento, y una etapa final de envejecimiento de la población. La primera etapa comenzó en Chile alrededor de 1900 y se extendió hasta principios de los años sesenta (figura 1). A partir de mediados de esta década, la caída de la tasa de natalidad en el país detuvo el crecimiento poblacional y este descenso, junto con una estabilización de las tasas de mortalidad, provocó una disminución del crecimiento. La reducción de la fertilidad asociada con una mayor esperanza de vida son los principales contribuyentes del envejecimiento poblacional (1–4). La esperanza de vida al nacer en Chile ha aumentado significativamente, pasando de 55 años en la década de los cincuenta a 78 años en la actualidad, y se espera que hacia 2100 se encuentre en torno a 90 años (5).

**FIGURA 2:**
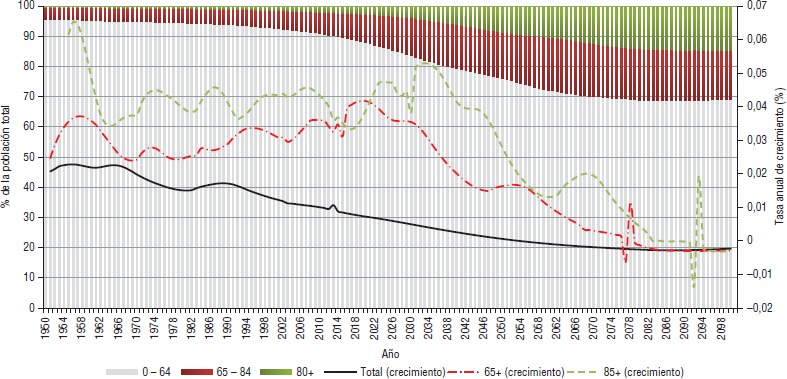
Evolución de la población de adultos mayores en Chile, porcentajes y crecimiento, 1950–2100

Chile está entrando en la última etapa de la transición demográfica. Como muestra la figura 2, la población mayor de 65 años, que ha aumentado a tasas crecientes durante los últimos 45 años, crecerá a una tasa mayor a 4% anual en los próximos seis años (2017–2023), lo que implicará que los adultos mayores de 65 años pasarán de ser 10% del total de los habitantes en 2010 a 20% en 2038 (periodo de 23 años). Por su parte, la población mayor de 80 años, que actual mente constituye cerca de 2,4% del total, crecerá a una tasa promedio anual de 4,5% durante los próximos 20 años y llegará a representar 5% de la población en 2035. Para 2100, las estimaciones indican que 30% de la población tendrá más de 65 años y que en este grupo de edad la mitad de las personas tendrá más de 80 años (5, 6).

El envejecimiento está asociado no sólo con un deterioro en el estado de salud físico de las personas, sino también con un importante aumento de la frecuencia de las enfermedades mentales. Ambos problemas —deterioro físico y mental— están directamente relacionados con el grado de dependencia de las personas. Tanto la prevalencia de dependencia como la de las enfermedades mentales aumentan de manera importante a partir de los 65 años y son particularmente prevalentes en los mayores de 85 años (7, 8). La carga de enfermedades mentales está usualmente subestimada (9) y es un factor importante que debe considerarse al estimar la dependencia de la población, ya que se espera que ésta aumente considerablemente en los próximos años. Por ejemplo, se estima que la prevalencia de la demencia en Chile se haya triplicado en 2050 (10). Los patrones mostrados para Chile son similares a los estimados para los países de la Organización para la Cooperación y el Desarrollo Económico (OCDE), donde las tasas de prevalencia de demencia se duplican cada lustro y las proyecciones indican que, en 20 años, el número de personas con demencia en este grupo de países también se duplicará (11).

***Fuente:*** Elaboración propia a partir de las referencias 5 y 6.

Lo anterior plantea un enorme desafío asociado con el envejecimiento, pero sobre todo un aumento del número de personas con pérdida de funcionalidad y algún grado de dependencia en el país. El objetivo de este documento es describir el escenario actual de los cuidados durante el envejecimiento a largo plazo en Chile, usando la experiencia de los países de la OCDE, para poner de manifiesto la necesidad de avanzar en el diseño y el financiamiento de una política coordinada en el país que permita afrontar con cierta antelación los desafíos del envejecimiento en las próximas décadas.

## Cuidados a largo plazo en Chile y la OCDE

Según las definiciones de la OCDE y de la Comisión Europea, se entiende por cuidados a largo plazo el conjunto de servicios requeridos por personas con un grado de funcionalidad física o cognitiva reducido que, por lo tanto, dependen de otra persona (durante un periodo largo de tiempo) para realizar sus actividades básicas de la vida diaria. Estos servicios incluyen actividades de cuidado personal, cuidados médicos básicos y ayuda en el hogar (12). A continuación, se presentan las principales acciones llevadas a cabo en Chile en la actualidad, así como un panorama de los sistemas de cuidados a largo plazo en los países de la OCDE.

### Administración y beneficios.

Actualmente, en Chile se desarrolla una serie de programas públicos en torno a cuidados a largo plazo implementados a través de dos ministerios: el de Salud (MINSAL) y el de Desarrollo Social (MDS). Desde el MINSAL las iniciativas se componen principalmente de programas para adultos mayores en el ámbito de la prevención y el tratamiento: acceso al Examen de Medicina Preventiva en el Adulto Mayor (EMPAM) y una atención primaria basada en sus resultados, detección de vicios de refracción y entrega de lentes ópticos, evaluación de necesidades y ayuda técnica y educación de su uso, hipoacusia bilateral, neumonía de atención ambulatoria y endoprótesis total de cadera para la artrosis de cadera y la limitación funcional grave. Además, el MINSAL desarrolla otras iniciativas, como programas de vacunación, el Programa de Alimentación Complementaria del Adulto Mayor (PACAM) y la entrega de lentes, audífonos, bastones y sillas de ruedas (13, 14).

En el MDS, las acciones son mayormente desarrolladas por el Servicio Nacional del Adulto Mayor (SENAMA). A pesar de tener una variada gama de iniciativas (15), más de 60% del presupuesto de esta institución se destina a financiar residencias para estancias de adultos mayores, a través del Fondo de servicios de atención al adulto mayor y el Fondo de subsidio para establecimientos de larga estadía (16). La mayor parte de los programas tienen criterios de inclusión de los beneficiarios, generalmente vinculados con la edad, el área de residencia y la condición socioeconómica. Evaluaciones recientes de algunos de estos programas muestran deficiencias en distintos niveles (cobertura, monitoreo y evaluación, gestión), que refuerzan la necesidad no sólo de coordinar iniciativas fragmentadas, sino, además, de rediseñar algunas intervenciones (17, 18).

***Fuente:*** Elaboración propia a partir de la referencias 5.

Por su lado, los cuidados a largo plazo en los países de la OCDE se incluyen generalmente en el sector salud. Buena parte de estos sistemas se basan en la existencia de dos alternativas para la prestación de servicios, aunque los países difieren en sus preferencias entre la prestación de los beneficios en forma de servicios (*in-kind*) o de subsidios monetarios (*in-cash*). La primera de estas dos alternativas corresponde a los cuidados en el hogar: servicios como asistencia en cuidados personales (ayuda para bañarse y vestirse), ayuda en labores del hogar (preparación de comidas), realización de actividades sociales, y servicios de enfermería y transporte. Y la segunda, a las instituciones de cuidados a largo plazo: derecho a usar servicios e instalaciones de las instituciones acreditadas para estos fines.

Tradicionalmente, los sistemas de cuidado a largo plazo han estado estrechamente ligados a instituciones especializadas como principal proveedor y servicios médicos como principal beneficio. Este paradigma se ha cuestionado en años recientes y en la actualidad los países de la OCDE han introducido reformas que buscan cambiar el foco de los cuidados a largo plazo, desde uno centrado en la atención médica a uno basado en la evaluación y el apoyo a la autosuficiencia (Alemania), separar la función médica de los cuidados a largo plazo (Corea del Sur y Suecia) e incentivar el uso de cuidados en el hogar sobre los cuidados en instituciones (Holanda y países nórdicos). Según las últimas estadísticas al respecto, cerca de 65% de la población mayor de 65 años en los países de la OCDE recibe cuidados a largo plazo en el hogar. A excepción de Finlandia, todos los países han avanzado en conceder más importancia a los cuidados en el hogar que a los cuidados prestados en instituciones (11). Según las últimas estadísticas al respecto, cerca de 65% de la población mayor de 65 años en los países de la OCDE13 recibe cuidados a largo plazo en el hogar (OCDE13 incluye a Hungría, Japón, Noruega, Suiza, Suecia, Holanda, Alemania, Corea del Sur, Francia, Finlandia, Luxemburgo, Australia y los Estados Unidos de América). A excepción de Finlandia, todos los países han avanzado en conceder más importancia a los cuidados en el hogar que a los cuidados prestados en instituciones (11).

En general, los sistemas de cuidado a largo plazo también incluyen beneficios para los cuidadores, como subsidios, permisos de ausencia laboral, entrenamiento y capacitación, y servicios de relevos (*respite care*) (19), lo cual refleja la necesidad de ayudar a una fracción cercana a 15% de las personas mayores de 50 años (especialmente mujeres) que reporta su actividad como cuidador en los países de la OCDE (11) y la importancia de la función que desempeñan como piedra fundamental del sistema (20).

La selección de beneficiarios y beneficios en estos países de la OCDE se realiza sobre la base de las necesidades detectadas usando algún instrumento para medir la dependencia. En el caso de Chile se emplea un instrumento validado (Evaluación Funcional del Adulto Mayor, EFAM-Chile), que se aplica en el Examen de Medicina Preventiva en el Adulto Mayor (EMPAM) para decidir las acciones preventivas de salud específicas que deben realizarse con cada persona. Lamentablemente, su cobertura es baja (en 2012 giraba en torno a 41,5%) y los criterios de asignación de beneficios difieren entre instituciones y programas (13–15).

### Financiamiento.

Como prioridades gubernamentales destacan dos hechos que expresan un mayor compromiso del país con los adultos mayores: la transformación del SENAMA en un servicio (pasando desde la Presidencia al MDS) y un aumento de presupuesto, que casi se duplicó entre 2012 y 2013. Sin embargo, a pesar de estos esfuerzos, la inversión en cuidados a largo plazo y las iniciativas dirigidas a los adultos mayores siguen siendo muy limitadas: el presupuesto del SENAMA para 2016 rondaba los US$29 millones cifra que corresponde a 0,07% del presupuesto del Gobierno Central. Las estimaciones realizadas indican que la puesta en marcha de un sistema de cuidados a largo plazo en Chile costaría alrededor de US$ 1 600 millones equivalentes a 0,45% del PIB (21). Actualmente, los países de la OECD están invirtiendo montos importantes en el financiamiento de sus sistemas de cuidados a largo plazo. Australia inyectó en 2015 US$ 1 600 millones al suyo (22). Alemania ha comprometido 5 000 millones de euros anuales adicionales para cuidados a largo plazo a partir de 2017 y actualmente gasta 1,5% de su PIB en esta área (23). Corea del Sur gasta 1,1% de su PIB, el Reino Unido, 1,8%, y Holanda, 3,0% (24).

A pesar de la creciente importancia del tema, se observa una alta heterogeneidad en el gasto en cuidados a largo plazo, incluso en los países de la OCDE, lo que refleja las distintas prioridades que estos países asignan a este problema. Lamentablemente, en Chile ni siquiera existen estadísticas al respecto, lo que también indica su baja relevancia en el país. Es posible apreciar, sin embargo, que la mayoría de los países ha ido aumentando su gasto en esta área, lo que se explica tanto por el constante envejecimiento de la población como por los cambios en las prioridades asignadas a este tema en el conjunto del sistema de salud del país (25). Por un lado, hay un grupo de países que en 2014 invertía más de 20% de su gasto en salud en cuidados a largo plazo: Holanda (27,2%), Dinamarca (22,1%), Islandia (20,4%), Noruega (25,8%) y Suecia (24,2%). Y, por otro, algunos países han decidido dar mayor prioridad al envejecimiento incrementando fuertemente su gasto: la República Checa pasó de gastar 4% de su gasto en salud en cuidados de largo plazo en 2013 a 12,4% en 2014; Estonia de 0,9% en 2001 a 3,6% en 2014; Japón de 7,8% en 2010 a 15,7% en 2011; Corea del Sur de 2,3% en 2006 a 11% en 2014; Holanda de 14% en 2004 a 24,7% en 2005, y España de 1,6% en 2002 a 5,3% en 2013 (24). Estos abruptos cambios no reflejan simplemente un gasto que crece a la par de la población, sino un cambio de política —avalado por mayor gasto— que reconoce la necesidad de hacer frente a una realidad del país.

Además de todo lo expuesto, un grupo de países, conscientes del desafío que supone financiar un sistema de cuidados a largo plazo en una sociedad que envejece, han implantado esquemas de financiamiento especiales para cubrir los gastos de su sistema. Actualmente, hay seis países en el mundo con un sistema de seguros obligatorios para cuidados a largo plazo: Holanda, Japón, Corea del Sur, Alemania, Israel y Luxemburgo (19). En el caso chileno, la idea de disponer de un seguro obligatorio parece ser una buena alternativa. En la esfera de la solidaridad, permite financiar los beneficios para un grupo vulnerable, independientemente de sus recursos y redes de apoyo, aumentando la cobertura del sistema (26); en la de la eficiencia, consigue un financiamiento especial para atender necesidades especiales y acotadas, manteniendo un mayor control y transparencia sobre los recursos en salud y evitando que el sistema de cuidados a largo plazo termine consumiendo los recursos destinados a prestaciones de salud (como actualmente ocurre con el pago de licencias médicas); en la de la sostenibilidad, logra disminuir el riesgo financiero respecto de sistemas financiados por el Estado y de sistemas de ahorro voluntario (21, 27); y, por último, en la esfera de la calidad, la existencia de un tercero —el seguro— permite controlar la calidad del servicio, un elemento especialmente importante en un mercado donde los pacientes tienen dificultades para evaluar y exigir la (28).

## CONCLUSIONES Y RECOMENDACIONES

El envejecimiento en Chile es una realidad: si bien el país aún no se encuentra en una etapa avanzada del envejecimiento poblacional en comparación con otros países desarrollados, se espera que la población mayor de 65 años crezca rápidamente en los próximos 20 años. Lo anterior es una moneda de dos caras: la situación actual (nivel) da la impresión que éste es un problema del cual no es necesario preocuparse ahora, mientras que la evolución demográfica (tasa de crecimiento) muestra que Chile no dispone de mucho tiempo para diseñar y desplegar un sistema de cuidados a largo plazo antes de que las necesidades se vuelvan apremiantes. El momento de debatirlo es ahora, cuando aún se puede actuar proactiva en lugar de reactivamente.

Existe la necesidad de diseñar e implementar un sistema coordinado de servicios, con un ente administrador claro, así como beneficios y beneficiarios bien definidos. En la actualidad, hay una serie de iniciativas fragmentadas, impulsadas por distintas instituciones, con distintos enfoques, prioridades y poblaciones. Es necesario implantar un sistema de cuidados a largo plazo coordinado, en el cual se consideren diversas alternativas en relación con la administración, los beneficios y el financiamiento. Como ilustran las experiencias de países como Alemania y Holanda, es importante evaluar la conveniencia de implantar una política centrada en el financiamiento de las instituciones de larga estadía en contraposición a un sistema enfocado a financiar cuidados en el hogar. Asimismo, se recomienda evaluar la implementación de un seguro obligatorio para cuidados a largo plazo como parte del sistema de la seguridad social del país.

Chile aún está a tiempo de iniciar un debate crucial de forma planificada. Es necesario incluir los cuidados a largo plazo y su financiamiento en la discusión de la reforma del sector salud, especialmente cuando el país ha empezado a reflexionar sobre su financiamiento y su grado de solidaridad. La implementación de un sistema de cuidados a largo plazo y su financiamiento, un tema hasta la fecha ignorado en la agenda pública, debe incluirse en la siguiente reforma del sistema, antes de que sea demasiado tarde.

## Agradecimiento.

Los autores agradecen los comentarios de distintos revisores anónimos, que contribuyeron a mejorar la versión final de este manuscrito. Todos los errores son exclusiva responsabilidad del autor.

## Financiación.

 Ninguna declarada por los autores.

## Declaración.

Las opiniones expresadas en este manuscrito son responsabilidad del autor y no reflejan necesariamente los criterios ni la política de la *RPSP/PAJPH* y/o de la OPS.
